# Differentiated instruction in Indonesian primary school physical education: a mixed-methods study

**DOI:** 10.3389/fspor.2026.1638892

**Published:** 2026-06-04

**Authors:** Wawan Sundawan Suherman, Yudik Prasetyo, Guntur Guntur, Cerika Rismayanthi, Beatrix J. Podung, Kamal Firdaus, Harry Pramono, Bambang Priyono, Ayi Suherman, Jonni Siahaan, Zaza Afnindar Fakhrurozi, Ade Gusti Ningsih, Amran

**Affiliations:** 1 Department of Sports Science, Universitas Negeri Yogyakarta, Yogyakarta, Indonesia; 2 Department of Physical Education, Universitas Negeri Yogyakarta, Yogyakarta, Indonesia; 3 Department of Sport Education, Universitas Negeri Manado, Tondano, Indonesia; 4 Department of Sport Education, Universitas Negeri Padang, Padang, Indonesia; 5 Department of Sport Education, Universitas Negeri Semarang, Semarang, Indonesia; 6 Department of Primary School Physical Education, Universitas Pendidikan Indonesia, Bandung, Indonesia; 7 Department of Physical Education, Universitas Singaperbangsa, Karawang, Indonesia; 8 Department of Primary School Physical Education, Universitas Negeri Yogyakarta, Yogyakarta, Indonesia; 9 Department of Primary Education, Universitas Negeri Yogyakarta, Yogyakarta, Indonesia

**Keywords:** differentiated instruction, elementary school students, instructional planning, physical education teacher, teaching implementation

## Abstract

Despite the growing global emphasis on differentiated instruction, empirical evidence regarding its implementation in primary school physical education within developing-country contexts remains limited. In Indonesia, differentiated instruction has been promoted through the Merdeka Curriculum; however, its practical implementation across diverse socio-cultural and geographical settings has not been comprehensively examined. This study investigates teachers’ understanding, instructional planning, implementation practices, and evaluation of differentiated instruction in Indonesian primary school physical education, while identifying the structural and contextual factors shaping its enactment. A sequential explanatory mixed-methods design was employed. Quantitative data were collected from 496 physical education teachers across seven Indonesian regions using a Likert-scale questionnaire analysed with SPSS version 25. The qualitative phase involved semi-structured interviews with 14 teachers selected through maximum variation sampling. Qualitative data were analysed thematically to enrich the interpretation of quantitative findings. The findings indicate that differentiated instruction remains unevenly implemented across regions. Approximately 41% of teachers demonstrated only moderate conceptual understanding, while 43% exhibited limited competence in differentiated lesson planning. Furthermore, 38% identified inadequate facilities and instructional resources as major barriers, and 78% reported that assessment practices remained only moderately adaptive. Qualitative findings further revealed pronounced regional disparities, limited professional development opportunities, uneven policy enactment, and restricted access to assessment tools. This study offers a novel contribution by extending Tomlinson’s differentiated instruction framework into the underexplored context of primary school physical education in a developing country characterised by significant socio-cultural and geographical diversity. The findings demonstrate that the effectiveness of differentiated instruction is strongly shaped by contextual realities, institutional support, and regional inequalities. The study highlights the need for context-responsive teacher education, differentiated assessment guidelines, and collaborative professional support systems to strengthen inclusive and adaptive pedagogical practices.

## Introduction

The 21st-century education paradigm emphasises learner-centred approaches, in which student is given opportunities to develop according to their potential, interests, and needs. In the context of physical education, this demand is particularly relevant because students exhibit diverse physical abilities, motivations, learning styles, and movement experiences. Differentiated learning, as conceptualised by Tomlinson (1), is regarded as a pedagogical approach that accommodates such heterogeneity by adjusting content, processes, products, and the learning environment. Its core principle highlights that student diversity is not an obstacle but rather a foundation for designing instruction that is equitable, effective, and inclusive (2, 3).

Globally, physical education is no longer seen merely as a vehicle for motor skill development, but also to foster movement literacy, health, and social character that supports lifelong active living (4–6). Recent studies confirm that adaptive strategies, including differentiated learning, enhance student engagement, motivation, and achievement (7, 8). International research provides strong evidence for the effectiveness of innovative pedagogical models. A systematic review by Elumalai et al. (9) showed that approaches such as Teaching Games for Understanding, project-based learning, and collaborative learning improve physical fitness, academic outcomes, and enjoyment among school-aged learners. Similarly, a meta-analysis by Dudley et al. (10), Synthesising evidence from 135 studies encompassing 42,500 participants, the review found that pedagogically centred approaches, including game-based and mastery teaching, significantly enhanced learning outcomes, particularly in psychomotor (*d* = 0.52) and affective (*d* = 0.47) domains. Further research emphasises modifying tasks and differentiating content to improve learning, as well as adopting hybrid models that integrate tactical and affective approaches (11–14). However, most of these studies were conducted in developed countries with robust infrastructure and relatively high teacher competence. Despite the breadth of international evidence, studies specifically examining differentiated learning in physical education remain limited. Akbaruddin et al. (15), for example, in a systematic review of 51 articles, identified only six that directly addressed the impact of differentiation on physical fitness and active lifestyles, signalling a critical research gap.

In Indonesia, differentiated instruction was formally acknowledged in the 2020 professional teacher education programme. It has since been positioned as a promising strategy that enables students to pursue individual learning trajectories while maintaining physical and mental well-being. Hasanah et al. (16) define differentiated learning as an approach that allows students to follow individual learning paths, rooted in the philosophy of progressive education, and responsive to readiness, interests, and learning profiles. Although it is theoretically effective in enhancing motivation, engagement, and achievement, its practice in Indonesian physical education remains limited. Empirical evidence on its implementation in this context is scarce (17). Maulana and Heynoek (18) note that differentiated learning in physical education is “limited and rarely practised by educators,” reflecting a substantial gap between policy and classroom realities. While it holds potential to promote sustainable education in Indonesian primary schools (19), teachers often rely on uniform, “one size fits all” strategies that overlook students' diverse physical, cognitive, and affective capacities. In relation to the *Merdeka Curriculum*, which promotes individualised, adaptive, and learner-centred approaches, teachers generally express positive attitudes towards differentiation but acknowledge challenges in practice (20). Research shows that its implementation in Indonesia is still largely descriptive, highlighting curriculum, practice gaps and barriers such as limited time, inadequate resources, and insufficient training (15, 18, 21–23). This raises key questions about teachers' capacity to interpret, plan, implement, and evaluate differentiated learning in large classes with limited facilities.

Further, primary schools in the Special Region of Yogyakarta, as partners of the Primary School Physical Education Study Programme, face persistent challenges in delivering Physical Education. Although evaluations report achievements in infrastructure, lesson tools, teaching processes, and learning outcomes, weaknesses remain in planning, teaching methods, assessment, and programme management (24–26). Despite its importance, differentiated learning in Physical Education has not reached its full potential. In practice, teachers sometimes base instruction on students' aspirations or talents, without recognising that each student possesses a distinct learning profile. This reinforces the persistent gap between ideal policy and classroom practice. Conceptually, differentiated learning offers a solution for student heterogeneity, yet in large, resource-limited classrooms it is often considered impractical. This raises a central controversy: is differentiated learning feasible in physical education within developing countries, or is it largely a normative ideal?

Existing studies underline a clear research gap: the lack of comprehensive inquiry into how Tomlinson's theory of differentiation is applied within the physical education ecosystem of Indonesian primary schools. Consequently, empirical studies that map teachers' understanding, planning, implementation, and evaluation of differentiated learning are urgently needed, both to strengthen classroom practice and to connect theory with national education priorities. Against this backdrop, the present study aims to analyse teachers' conceptual understanding, planning strategies, implementation practices, and evaluation approaches in Indonesian primary school physical education, using a mixed-methods design. This approach allows exploration of the extent to which differentiation has been practised, the challenges encountered, and opportunities for developing more adaptive pedagogies that respond to student diversity. The contribution of this study is twofold. Theoretically, it extends international literature on Tomlinson's framework by situating it within the under-researched context of physical education in developing countries. Practically, it provides empirical insights into Indonesian classroom realities, offering an evidence base for policy, teacher training programmes, and more inclusive and equitable pedagogical innovations.

## Methods

### Research design

**Figure 1:**
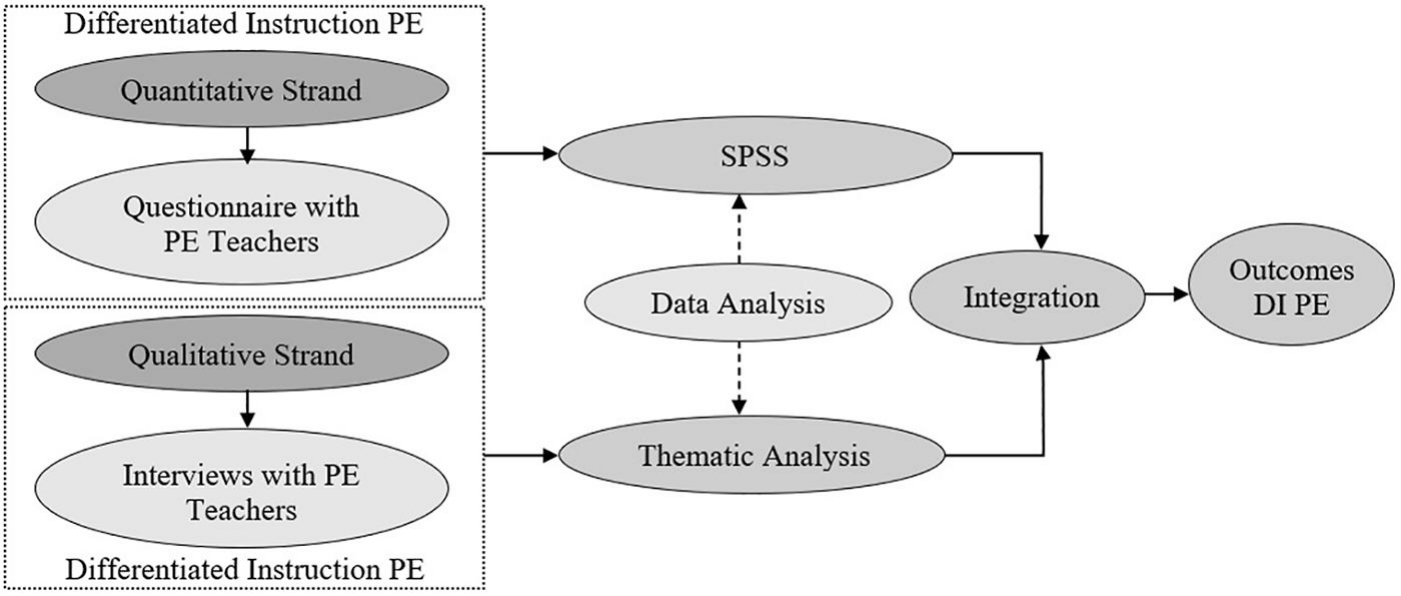
Research design flow.

This study adopted a sequential explanatory mixed-methods design, integrating quantitative and qualitative approaches in a phased and complementary manner (27–29). This design was particularly appropriate for developing a nuanced and multi-layered understanding of teachers' conceptualisation, planning, implementation, and evaluation of differentiated instruction in primary school physical education in Indonesia. The study was conducted in two interconnected phases. The quantitative phase involved a large-scale survey to identify general patterns and trends in teachers' practices. This was followed by a qualitative phase, in which semi-structured interviews were conducted to further explain, contextualise, and deepen the interpretation of the quantitative findings. This sequential structure enabled methodological complementarity, whereby statistical patterns were enriched with in-depth, context-sensitive insights. Figure 1 illustrates the sequential explanatory mixed-methods design employed in this study, integrating quantitative and qualitative phases.

### Population and sample

The target population comprised all primary school Physical Education (PE) teachers across Indonesia. Given the country's geographical vastness, regional disparities, and logistical constraints, a purposive non-probability sampling strategy was employed.

This approach was selected to ensure the inclusion of participants with direct relevance to the research focus, particularly teachers who were actively engaged in implementing the Merdeka Curriculum and exposed to differentiated instruction practices. The inclusion criteria were: (1) active primary school PE teachers, and (2) teachers involved in curriculum implementation.

Recognising the limitations of non-probability sampling, several strategies were implemented to mitigate sampling bias and enhance representativeness. First, data collection was conducted through a national-level professional forum (National Workshop for PE Teachers), which enabled access to participants from diverse geographical regions. Second, respondents were drawn from all major Indonesian regions, including Sumatra, Java, Kalimantan, Sulawesi, Nusa Tenggara, Maluku, and Papua. Third, variation in teaching experience, school context, and regional characteristics was deliberately considered during participant recruitment to ensure heterogeneity.

In the quantitative phase, a total of 496 teachers participated in the survey. While the sampling design does not support statistical generalisation, the study aims for analytical generalisation, whereby findings reflect patterns across diverse contexts rather than representing the entire population in a probabilistic sense.

For the qualitative phase, 14 participants were selected using a maximum variation purposive sampling technique, ensuring representation across Indonesia's three time zones (Western, Central, and Eastern) and multiple provincial contexts. This strategy enhanced the richness and diversity of perspectives captured. The sample size was considered sufficient to achieve thematic saturation and analytical depth (30, 31).

### Research instruments

#### Quantitative stage

The quantitative data were collected using a structured questionnaire administered via Google Forms. The instrument measured four core dimensions of differentiated instruction: understanding, planning, implementation, and evaluation, which are widely recognised in the literature on differentiated pedagogy. The questionnaire was adapted from the Differentiated Instruction Questionnaire (DI-Quest) developed by Coubergs et al. (32), with contextual modifications to ensure relevance to Indonesian primary education. Each item was explicitly aligned with indicators within the four dimensions, ensuring construct validity and conceptual coherence. Responses were measured using a four-point Likert scale (1 = Strongly Disagree to 4 = Strongly Agree), allowing for the assessment of both teachers' perceptions and self-reported instructional practices (33–35).

#### Qualitative stage

Qualitative data were generated through semi-structured interviews, conducted via Zoom or WhatsApp Call, with each session lasting approximately 30–45 min. The interview protocol was designed to extend and deepen the quantitative findings. Importantly, the interview guide was structured around the same four dimensions, understanding, planning, implementation, and evaluation, ensuring conceptual alignment and integration across both research phases.

### Instrument validity and reliability

The instruments used in this study were derived from established frameworks and adapted to ensure contextual appropriateness.

For the quantitative instrument, the DI-Quest (32) underwent a forward–backward translation process to ensure semantic equivalence. A pilot study involving five PE teachers was conducted to assess clarity and relevance. The instrument has demonstrated strong psychometric properties, including acceptable construct validity (CFI = 0.919; TLI = 0.911; RMSEA = 0.041; SRMR = 0.048) and internal consistency (*α* = 0.632–0.858).

For the qualitative instrument, the interview guide was adapted from the Instrument to Assess Teachers' Practice of Differentiated Instruction (IATPDI) (36). Content validity was established using a Delphi techniqueinvolving two national experts. The results indicated high agreement (I-CVI = 1.00 for 97.5% of items; S-CVI/UA = 0.98; modified Kappa = 1.00), confirming the instrument's validity.

### Data analysis techniques

#### Quantitative data analysis

Quantitative data were analysed using SPSS, employing both descriptive and inferential statistical techniques (37, 38). Descriptive statistics (mean, standard deviation, minimum, and maximum) were used to determine the levels of teachers' understanding, planning, implementation, and evaluation of differentiated instruction. To enhance interpretability, scores were categorised into five levels: Very High, High, Moderate, Low, and Very Low, based on standard deviation from the mean (Table 1).

**Table 1:** Five-category classification framework.

Interval	Category
M + 1.5SD < X	Very high
M + 1.5SD < X ≤ M + 0.5SD	High
M + 0.5SD < X ≤ M − 0.5SD	Medium
M − 0.5SD < X ≤ M − 1.5SD	Low
X ≤ M − 1.5SD	Very low

#### Qualitative data analysis

Qualitative data were analysed using thematic analysis, following the six-phase framework proposed by Braun and Clarke (39). This process involved transcription, initial coding, theme generation, theme refinement, and interpretive synthesis (40–42). This analytical approach enabled a rich, contextualised interpretation of teachers' experiences, practices, and challenges in implementing differentiated instruction across diverse Indonesian educational settings.

### Data integration

The findings are presented in accordance with the sequential research design, integrating quantitative and qualitative evidence for each core dimension to generate comprehensive and contextually grounded interpretations.

## Results

The results are presented in alignment with the research stages, integrating quantitative and qualitative findings for each core variable to generate meaningful and contextually relevant interpretations.

### Descriptive statistics

Based on the analysis conducted using SPSS Version 25, descriptive statistics for the 496 valid responses are summarised in Table 2. The analysis focuses on four principal dimensions of differentiated instruction: understanding, planning, implementation, and evaluation. Collectively, these dimensions provide an overarching representation of differentiated instructional practices in primary school physical education across Indonesia.

**Table 2:** Descriptive statistics of differentiated learning of Indonesian physical education.

Descriptive statistics						
	*N*	Range	Minimum	Maximum	Mean	Std. deviation
Understanding differentiated physical education learning	496	48.00	16.00	64.00	488.065	532.223
Differentiated physical education learning planning	496	30.00	10.00	40.00	286.532	369.558
Implementation of differentiated physical education learning	496	51.00	17.00	68.00	540.665	594.670
Evaluating differentiated physical education learning	496	45.00	15.00	60.00	427.762	515.265

The descriptive statistics in Table 2 reveal discernible variation across the four dimensions. Scores for understandingranged from 16 to 64 (*M* = 48.81, SD = 5.32), while planning ranged from 10 to 40 (*M* = 28.65, SD = 3.70). For implementation, scores varied between 17 and 68 (*M* = 54.07, SD = 5.95), whereas evaluation ranged from 15 to 60 (*M* = 42.78, SD = 5.15). These findings indicate moderate dispersion, suggesting variability in teachers' engagement with differentiated instruction.

To further refine the interpretation of these quantitative findings, follow-up interviews were conducted with teachers representing both the highest and lowest scoring groups. Fourteen PE teachers were purposively selected to ensure geographical and contextual diversity (Table 3). This qualitative phase enriched the statistical findings by providing deeper insights into contextual practices and constraints.

**Table 3:** List of interview participants.

No.	Region	Teacher identity	School location
1	Sumatera	Teacher A	North Sumatra
2	Sumatera	Teacher B	West Sumatra
3	Jawa	Teacher A	Kebumen, Central Java
4	Jawa	Teacher B	Surabaya, East Java
5	Sulawesi	Teacher A	Gowa, South Sulawesi
6	Sulawesi	Teacher B	Kendari, South-East Sulawesi
7	Papua	Teacher A	Jayapura, Papua
8	Papua	Teacher B	Merauke, Papua
9	East Nusa Tenggara	Teacher A	Kupang, NTT
10	East Nusa Tenggara	Teacher B	Ende, NTT
11	Kalimantan	Teacher A	Pontianak, West Kalimantan
12	Kalimantan	Teacher B	Balikpapan, East Kalimantan
13	Bali	Teacher A	Denpasar, Bali
14	Bali	Teacher B	Gianyar, Bali

### Understanding of differentiated instruction

Building on the overall descriptive results, the first dimension examined is teachers' understanding of differentiated instruction. The analysis revealed a minimum score of 16 and a maximum of 64, with a mean of 48.8 (SD = 5.3). To enhance interpretability, scores were categorised into five levels: very high, high, moderate, low, and very low (Table 4).

**Table 4:** Understanding of differentiated learning in physical education.

Interval	Category	Total	Percentage
X > 57	Very high	40	8%
51 < X ≤ 57	High	84	17%
46 < X ≤ 51	Moderate	204	41%
41 < X ≤ 46	Low	155	31%
X ≤ 41	Very low	13	3%
Total		496	100%

The distribution indicates that most teachers fall within the moderate (41%) and low (31%) categories, while fewer demonstrate high (17%) and very high (8%) levels. A small proportion (3%) remains at a very low level. These findings suggest that teachers possess a basic conceptual awareness, but lack deeper pedagogical understanding. This pattern may be attributed to limited exposure to formal training and insufficient theoretical grounding in differentiated instruction.

To further elucidate these quantitative patterns, qualitative findings (Table 5) reveal that most teachers interpret differentiation primarily as adjusting teaching methods to accommodate students' abilities and physical conditions. For instance:

**Table 5:** Summary of thematic findings: primary school PE teachers’ understanding of differentiated instruction.

No	Location (province)	Key understanding of differentiated learning	Emphasised physical education context	Knowledge source	Key challenges
1	Teacher A North Sumatra	Adjusting to students’ needs, interests, and abilities	Difficult due to tools and time	Not mentioned	Tools & time
2	Teacher B West Sumatra	One method does not suit all students	Differences in learning speed	Personal understanding	Not mentioned
3	Teacher A Jawa Tengah	Approaches tailored to abilities and learning styles	Variations in students’ motor skills	Not mentioned	Not mentioned
4	Teacher B East Java	Familiar from independent curriculum training	Differences in physical condition	Independent Curriculum Training	Not mentioned
5	Teacher A South Sulawesi	Providing space according to each student's abilities	Physical strength & coordination	Not mentioned	Not mentioned
6	Teacher B South-East Sulawesi	Adjusting to students’ conditions	Nutrition & limited tools	Independent Curriculum Education and Training	Nutrition & facilities
7	Teacher A Papua (Jayapura)	Tailored to individual abilities and needs	Variety in physical readiness	Not mentioned	Nutrition & facilities
8	Teacher B Papua (Merauke)	Materials and methods tailored to students’ needs	Differences in motivation & physical condition	Not mentioned	Tools & nutrition
9	Teacher A NTT (Kupang)	Adjusting to students’ physical and psychological conditions	Physical & psychological diversity	Not mentioned	Tools & time
10	Teacher B NTT (Ende)	Methods and materials tailored to student characteristics	Prevent students from falling behind	Not mentioned	Tools & time
11	Teacher A Kalimantan (Pontianak)	Materials/methods tailored to each student's abilities	Optimise learning	Not mentioned	Not mentioned
12	Teacher B Kalimantan (Balikpapan)	Tailored to student needs and characteristics	Variations in the classroom	Not mentioned	Tools & time
13	Teacher A Bali (Denpasar)	Strategies tailored to individual needs and abilities	Optimise student development	Not mentioned	Not mentioned
14	Teacher B Bali (Gianyar)	Activities tailored to student characteristics	All students can develop	Not mentioned	Not mentioned

“I realise that one method cannot be applied to all students… there must be adjustments in teaching motor skills.”

This reflects an emergent awareness of learner diversity; however, the strategies described remain relatively basic. Structural constraints further complicate implementation. A teacher from Southeast Sulawesi noted:

“Some children are malnourished and equipment is limited, making it difficult to apply different strategies.”

Conversely, teachers who had participated in Merdeka Curriculum training demonstrated more operationalised understanding. A teacher from East Java explained:

“I learned about differentiated learning from the Merdeka Curriculum training. It helped me to try to adjust activities to the physical condition of students, for example by providing variations in the level of difficulty of movements.”

Taken together, the findings indicate that conceptual understanding is developing but not yet fully internalised, with formal training emerging as a critical enabling factor. Overall, the quantitative and qualitative findings converge. The survey shows that most teachers possess only a basic conceptual grasp, while interviews confirm that they struggle to apply differentiation due to limited resources, time, and diverse student needs. Teachers with access to structured training, however, are more likely to operationalise differentiated practices.

### Planning of differentiated instruction

Following the analysis of understanding, the next dimension concerns teachers’ capacity to design differentiated instruction.

The analysis yielded a mean score of 28.65 (SD = 3.70), with scores ranging from 10 to 40. The categorical distribution (Table 6) shows that most teachers are classified as low (43%) and moderate (31%), while fewer reach high (18%) or very high (6%) levels. These results indicate that differentiated planning is not yet systematically embedded in instructional design, likely due to constraints such as limited instructional time, large class sizes, and restricted access to pedagogical resources.

**Table 6:** Differentiated instruction planning for physical education.

Interval	Category	Total	Percentage
X > 34	Very high	32	6%
30 < X ≤ 34	High	89	18%
27 < X ≤ 30	Moderate	154	31%
23 < X ≤ 27	Low	211	43%
X ≤ 23	Very low	10	2%
Total		496	100%

To provide further explanatory depth, qualitative findings (Table 7) highlight that teachers attempt to incorporate differentiation through alternative activities and varying levels of task difficulty. However, these efforts are frequently constrained. As one teacher explained:

**Table 7:** Thematic differentiated instruction planning.

Main theme	General description	Representative quote	Region
Alternative activities based on student abilities	Most teachers develop lesson plans that include activities with varying levels of difficulty.	“*I plan by providing alternative activities… I give options to adjust the duration and intensity of the exercises*.”	South-East Sulawesi
		“*I plan my lesson plans by providing several alternative physical activities*…”	Central Java
Adjustment to facilities/Infrastructure	Teachers consider limitations in tools, facilities, and the number of students when planning lessons.	“*…but this is not always possible because there are too many students*.”	North Sumatra
		“*I also pay attention to the school environment and the limitations of sports equipment…*”	NTT
Traditional games & local culture	Teachers try to incorporate traditional games to make learning more interesting and contextual.	“*I also try to incorporate traditional games to make it more interesting for students*.”	Papua (Merauke)
		“*I also integrate local cultural elements to make learning more meaningful*.”	Bali (Gianyar)
Variations in activity levels	Teachers design activities with different levels (easy, medium, difficult) so that all students can participate.	“*I design three levels of activities…children can choose according to their abilities*.”	East Java
		“*I create plans with several options, from easy to challenging*.”	NTT (Ende)
Considerations of learning styles	Some teachers are beginning to pay attention to differences in learning styles when designing lessons.	“*I also consider the variety of students’ learning styles*.”	Bali (Denpasar)

“I provide alternative activities… but it is difficult due to large class sizes and limited time”

Similarly, a teacher from East Nusa Tenggara emphasised infrastructural limitations:

“Not all planned activities can be implemented due to limited equipment.”

Despite these challenges, a subset of teachers demonstrated innovative practices, including the integration of traditional games and culturally relevant strategies. These approaches suggest that effective planning is closely associated with contextual adaptation and pedagogical creativity. Overall, the findings highlight a systemic gap between pedagogical awareness and practical planning capacity, largely shaped by structural constraints.

### Implementation of differentiated instruction

Building on the planning dimension, the implementation phase reflects how differentiated strategies are enacted in classroom practice.

The analysis shows a mean score of 54.07 (SD = 5.95), with scores ranging from 17 to 68. The distribution (Table 8) indicates that most teachers fall within the low (38%) and moderate (33%) categories, while fewer achieve high (21%) or very high (7%) levels. This suggests that the implementation of differentiated instruction remains inconsistent and only partially realised.

**Table 8:** Implementation of differentiated instruction in physical education.

Interval	Category	Total	Percentage
X > 63	Very high	34	7%
57 < X ≤ 63	High	103	21%
51 < X ≤ 57	Moderate	165	33%
45 < X ≤ 51	Low	189	38%
X ≤ 45	Very low	5	1%
Total		496	100%

To further explain these findings, qualitative evidence (Table 9) indicates that teachers employ various adaptive strategies, including flexible grouping, peer teaching, and student choice. For example:

**Table 9:** Thematic implementation of physical education differentiated instruction learning in primary school.

Theme	Sub-theme	Key quote	Challenges faced
Student grouping	Based on physical ability	“*I usually divide students into groups based on their physical abilities*.” (Teacher A, North Sumatra)	Lack of assistants (Teacher A, North Sumatra; Teacher A, Jawa Tengah)
	Based on student readiness	“*They choose activities according to their readiness*.” (Teacher B, West Sumatra)	Short physical education lessons (Teacher B, West Sumatra; Teacher A, Kalimantan)
	Group rotation	“*I rotate so that everyone tries different levels*.” (Teacher B, East Java)	Large classes without teaching assistants (Teacher A, Jawa Tengah)
Learning strategies	Different instructions for each group	“*I give different instructions so that each group can follow*.” (Teacher A, Papua)	Limited facilities (Teacher A, South Sulawesi; Teacher B, Papua; Teacher A, NTT)
	Peer teaching	“*More proficient students help their friends*.” (Teacher B, South-East Sulawesi; Teacher B, Papua)	Insufficient sports equipment (Teacher A, Papua; Teacher A, NTT)
	Freedom to choose activities	“*I give students freedom of choice*.” (Teacher B, East Java)	Limited time and resources (Teacher A, South Sulawesi; Teacher A, Bali)
Learning atmosphere	Inclusive & confident	“*They are not ashamed to show their abilities*.” (Teacher B, East Java)	Creativity is needed (Teacher B, Papua Teacher B, Kalimantan)
	Motivation & active participation	“*I encourage them to choose activities that suit them, not to compare themselves with others*.” (Teacher B, South-East Sulawesi)	Use of local materials (Teacher B, Papua; Teacher B, NTT)

“I divide students based on their physical abilities…”

However, these practices are frequently limited by contextual challenges such as large class sizes, lack of teaching assistants, and insufficient facilities. Another teacher highlighted time constraints:

“Students are given choices, but limited time restricts deeper evaluation.”

Here, differentiation enhances motivation but remains constrained by insufficient instructional time.

Peer teaching emerged as a pragmatic strategy, particularly in resource-limited contexts. However, its effectiveness depends on student readiness and teacher facilitation. Importantly, teachers also emphasised the creation of inclusive learning environments that foster confidence and participation. This underscores that differentiation extends beyond instructional techniques to encompass affective and motivational dimensions. Overall, implementation reflects a partial and adaptive application of differentiation, shaped by both pedagogical intent and structural limitations.

### Evaluation of differentiated instruction

The final dimension examined is the evaluation of differentiated instruction.

The analysis revealed a mean score of 42.78 (SD = 5.15), with scores ranging from 15 to 60. The categorical distribution (Table 10) shows that most teachers fall within the moderate (43%) and low (35%) categories, while fewer reach high (13%) or very high (7%) levels.

**Table 10:** Evaluating differentiated instruction of physical education.

Interval	Category	Total	Percentage
X > 57	Very high	36	7%
51 < X ≤ 57	High	63	13%
46 < X ≤ 51	Moderate	211	43%
41 < X ≤ 46	Low	176	35%
X ≤ 41	Very Low	10	2%
Total		496	100%

These findings indicate that differentiated assessment practices remain underdeveloped and inconsistently applied.

Qualitative findings (Table 11) provide critical insights into this pattern. Many teachers continue to rely on standardised assessments, as reflected in the following statement:

**Table 11:** Thematic evaluation of physical education differentiated instruction learning in primary school.

No	Teachers & locations	Evaluation method	Key quotes	Key challenges
1	Teacher A North Sumatra	Standardised tests	“*Still using one test for all, currently learning to create a flexible rubric*.”	Lack of differentiation rubrics
				Limited time for individual observation.
2	Teacher B West Sumatra	Observation of attitude and cooperation	“*Different evaluation: physical test + attitude observation*.”	Long observation time
				Subjectivity of affective assessment.
3	Teacher A Jawa Tengah	Individual observation	“*Observation format for effort, cooperation, and courage to try*.”	Complex documentation for large classes
				Need for standardised tools.
4	Teacher B East Java	Holistic rubric	“*Rubric includes participation and affective aspects, not just movement results*.”	Time-consuming preparation of rubrics
				Assessment criteria must be clear.
5	Teacher A South Sulawesi	Skill tests + observation of attitude	“*Value cooperation and sportsmanship, not just skills*.”	Imbalance in focus (attitude vs. skills).
6	Teacher B South-East Sulawesi	Observation journal + rubric	“*Use personal journals and discussions with homeroom teachers*.”	Collaboration with class teachers is not always easy.
				Need for consistency in documentation.
7	Teacher A Papua (Jayapura)	Observation of development	“*Grades based on effort and attitude, not just technical skills*.”	Limited facilities affect assessment variation.
8	Teacher B Papua (Merauke)	Observation + recording of progress	“*Discuss with homeroom teachers for a complete picture*.”	Limited recording tools (e.g., video).
9	Teacher A NTT (Kupang)	Direct feedback	“*Observe physical development + provide feedback to motivate*.”	Students are less open to criticism.
10	Teacher B NTT (Ende)	Observation + student feedback	“*Ask students to express their difficulties*.”	Students are shy or unaccustomed to reflection.
11	Teacher A Kalimantan (Pontianak)	Observation + motivation to participate	“*Provide regular feedback to engage students*.”	Limited lesson time for in-depth feedback.
12	Teacher B Kalimantan (Balikpapan)	Reflection with students	“*Identify students’ difficulties through discussion*.”	Not all students are active in reflection.
13	Teacher A Bali (Denpasar)	Personal feedback	“*Provide personal feedback so students feel valued*.”	Increased workload for teachers.
14	Teacher B Bali (Gianyar)	Recognition of effort	“*Appreciate those who try hard*.”	The criteria for ‘hard work’ are difficult to measure objectively.

“I still use one standard test for all students…”

Nevertheless, some teachers have begun to adopt more holistic approaches, incorporating participation, effort, and attitude into assessment criteria:

“The rubric includes participation and affective aspects…”

In resource-constrained contexts, particularly in eastern Indonesia, evaluation practices rely heavily on observation and feedback rather than formalised instruments. Innovative approaches, such as the use of observation journals and collaboration with homeroom teachers, were also identified. However, these practices remain inconsistent and often depend on individual initiative. Overall, the findings reveal a significant gap between assessment theory and classroom practice, with systemic constraints limiting the full implementation of differentiated evaluation.

Taken together, the integration of quantitative and qualitative findings reveals a consistent pattern across all four dimensions. While teachers demonstrate an emerging awareness of differentiated instruction, its implementation remains partial, uneven, and context-dependent. The predominance of moderate and low categories reflects systemic challenges, including limited resources, large class sizes, insufficient professional development, and constrained instructional time. Conversely, teachers who demonstrate higher levels of implementation tend to exhibit innovative, context-sensitive practices, often supported by access to training and professional learning opportunities. These findings underscore the need for systemic interventions, including targeted professional development, improved resource allocation, and institutional support, to facilitate the effective implementation of differentiated instruction in primary school physical education.

## Discussion

This study provides a comprehensive examination of differentiated instruction (DI) practices in primary school physical education in Indonesia, revealing a consistent pattern across the four dimensions of understanding, planning, implementation, and evaluation. While the findings confirm that teachers demonstrate an emerging awareness of differentiation principles, the overall pattern indicates that DI remains only partially implemented and insufficiently institutionalised. This discrepancy points to a systemic gap between policy aspirations and classroom realities.

From a theoretical perspective, this gap can be interpreted through the lens of policy implementation theory and teacher readiness frameworks. Although the Merdeka Curriculum explicitly promotes differentiated, student-centred learning, the translation of these principles into practice depends on teachers' pedagogical capacity, institutional support, and contextual resources. The findings suggest that these enabling conditions are unevenly distributed, resulting in fragmented and inconsistent implementation across regions.

### Understanding differentiated instruction in physical education

The findings indicate that physical education teachers' understanding of differentiated instruction predominantly falls within the *moderate to low* categories, with a mean score of 48.8 out of 64. Qualitative evidence further suggests that teachers generally conceptualise differentiation as the adaptation of content, process, and product to accommodate students' readiness, interests, and learning profiles. This interpretation is consistent with Carol Ann Tomlinson's framework, which positions differentiation not merely as a set of instructional techniques but as an inclusive pedagogical philosophy that recognises learner diversity as an asset (1). It also aligns with the Merdeka Curriculum's emphasis on teacher flexibility, reflective practice, and adaptive competence within the broader Merdeka Belajar agenda (43–45).

However, the persistence of moderate and low levels of understanding suggests a critical gap between conceptual awareness and pedagogical internalisation. This gap can be attributed to several interrelated factors. First, professional development related to differentiated instruction remains uneven in both access and quality, limiting teachers' opportunities to translate theoretical knowledge into actionable classroom strategies. Second, institutional support mechanisms—such as mentoring systems, instructional guidelines, and reflective supervision—are not yet systematically embedded at the school level. Third, entrenched instructional cultures, which often prioritise uniformity and standardisation, may inhibit the adoption of more flexible and student-centred approaches.

In international contexts, similar patterns have been observed. In Turkey, differentiated learning has been shown to enhance student engagement and performance, yet teachers encounter significant practical constraints (46, 47). Studies in Ukraine report improved participation and efficiency when differentiation is implemented systemically (48), while research from China and the Netherlands highlights teacher adaptability as a decisive factor (7, 49). Across these contexts, the effectiveness of differentiation is consistently linked to sustained training, adequate resources, and coherent institutional support (48, 50).

Importantly, this study reveals pronounced regional disparities within Indonesia. Teachers in western regions (e.g., Sumatra and Java) demonstrate relatively stronger integration of holistic assessment practices, whereas those in eastern regions (e.g., Papua and NTT) rely more heavily on informal strategies such as verbal feedback and observational judgement. These differences are not solely pedagogical but reflect broader structural inequalities, including disparities in infrastructure, access to training, and socio-economic conditions.

Consequently, the gap between policy and practice can be understood as a function of *policy enactment failure*, where national curriculum ideals are not fully translated into localised practice due to uneven resource distribution and varying levels of teacher readiness. Addressing this issue requires multi-level interventions, including sustained capacity-building programmes, targeted support for under-resourced regions, and the development of context-sensitive instructional and assessment tools. Strengthening collaboration among schools, local governments, and communities is also essential to ensure that the inclusive principles of the Merdeka Curriculum are realised in practice.

### Planning differentiated instruction in physical education

The findings indicate that the quality of differentiated instructional planning remains predominantly within the *low to moderate* categories, with only a small proportion of teachers demonstrating high-level competence. Despite this, qualitative data reveal that some teachers have begun to incorporate differentiation principles by offering alternative activities, adapting to resource constraints, and acknowledging students' diverse learning profiles and cultural contexts. This suggests the presence of *incipient pedagogical awareness* that has not yet been systematically translated into structured planning practices.

According to Tomlinson (1), effective differentiated planning requires a comprehensive understanding of learner variability, the formulation of adaptive learning objectives, and the implementation of evidence-based instructional strategies. However, the findings suggest that these elements are not yet consistently operationalised in the Indonesian context. This limitation can be explained by several structural and systemic factors. First, high teacher–student ratios reduce the feasibility of designing and implementing differentiated lesson plans. Second, limited access to facilities and instructional resources constrains teachers' ability to diversify learning activities. Third, geographical disparities create uneven access to professional development, further reinforcing differences in planning quality.

Similar challenges have been documented in other Global South contexts, including Pakistan, the Maldives, and Ethiopia, where differentiated planning tends to be sporadic and largely dependent on individual teacher initiative rather than institutionalised support systems (51, 52). This suggests that the issue is not merely individual but systemic, reflecting broader limitations in policy implementation and educational infrastructure.

Within the framework of the Merdeka Curriculum, which explicitly promotes adaptive learning and the development of Pancasila learner profiles, these findings indicate that differentiation has not yet been fully internalised as a professional norm. Although the policy provides conceptual guidance, the absence of practical implementation frameworks, such as model lesson plans, scaffolding tools, and contextual guidelines, limits its effectiveness at the classroom level (53, 54).

Regional disparities further reinforce this gap. Teachers in western Indonesia, benefiting from relatively better access to training and infrastructure, are more likely to demonstrate systematic planning practices. In contrast, teachers in eastern regions often rely on culturally embedded strategies, such as traditional games. While these approaches enhance contextual relevance and student engagement, they remain insufficiently integrated into formal pedagogical frameworks. This indicates the need for an *asymmetric policy approach* that prioritises disadvantaged regions through targeted investment, capacity-building, and resource redistribution.

When compared with developed education systems, the contrast becomes more pronounced. In Finland, differentiation is embedded within the national curriculum and supported by strong professional autonomy and a culture of reflective practice (55, 56). Similarly, in Norway, differentiation is reinforced through collaborative planning, professional supervision, and data-informed decision-making (57, 58). By contrast, in many Global South contexts, including Indonesia, planning remains fragmented and insufficiently institutionalised (59).

Overall, the findings suggest that the gap between policy and practice in differentiated planning is driven by a combination of limited teacher readiness, insufficient structural support, and uneven policy implementation. Addressing this gap requires not only strengthening teacher competencies but also developing practical, context-sensitive planning frameworks that can be feasibly implemented across diverse educational settings.

### Implementing differentiated instruction in physical education

The findings indicate that the implementation of differentiated instruction in Indonesian primary school physical education remains largely within the *low to moderate* categories, with a mean score of 54.1. This pattern reflects inconsistency in translating pedagogical understanding into classroom practice. Although qualitative data reveal that teachers employ adaptive strategies—such as ability-based grouping, flexible activity selection, and peer-assisted learning—these practices remain fragmented and situational rather than systematically embedded.

From a theoretical perspective, effective differentiation requires the deliberate adaptation of content, process, product, and learning environment to align with students' diverse needs (1). However, the findings suggest that teachers' practices are predominantly intuitive, lacking the structural and pedagogical coherence necessary for sustained implementation. This discrepancy can be explained through several interrelated factors.

First, resource constraints significantly limit implementation capacity. The absence of adequate facilities, insufficient teaching aids, and large class sizes reduce the feasibility of managing multiple differentiated activities simultaneously. This aligns with findings by Colquitt et al. (60), which emphasise that physical education contexts require substantial logistical support to implement differentiation effectively.

Second, time constraints within the curriculum structure further restrict teachers' ability to operationalise differentiation. Physical education sessions are often limited in duration, prioritising activity completion over reflective and adaptive teaching processes. Consequently, differentiation tends to be reduced to surface-level modifications rather than deeper pedagogical transformations.

Third, teacher readiness and professional support systems remain uneven. While teachers demonstrate awareness of differentiation principles, the absence of continuous mentoring, classroom-based coaching, and reflective supervision inhibits the development of more sophisticated instructional practices. This reflects a broader issue of *policy enactment*, where curriculum mandates are not fully translated into practice due to limited institutional scaffolding.

These findings are consistent with evidence from other Global South contexts. In Eritrea, teachers reported relying on intuition due to a lack of formal training (61). In Pakistan, implementation barriers included limited institutional support, constrained instructional time, and weak school leadership (62). Similarly, in South Africa, high levels of conceptual awareness did not translate into effective practice due to inadequate training and systemic constraints (63). Across these contexts, differentiation often remains confined to minor instructional adjustments rather than comprehensive, evidence-based strategies (15, 60, 64, 65).

Within the Merdeka Curriculum, differentiation is positioned as a central principle of student-centred learning. However, the findings suggest that its implementation is constrained by the absence of *operational mechanisms*, such as structured teaching modules, differentiated task banks, and context-sensitive instructional guidelines. Without these supports, teachers are required to independently interpret and enact policy expectations, leading to variability in practice.

Geographical disparities further exacerbate these challenges. Teachers in western Indonesia, who benefit from relatively better access to training and digital resources, demonstrate greater capacity to implement differentiated strategies. In contrast, teachers in eastern regions rely more heavily on adaptive practices rooted in local contexts, such as traditional games and peer teaching. While these approaches enhance engagement, they are not yet systematically integrated into formal pedagogical frameworks. Comparable findings in the Maldives indicate that differentiation is often simplified into static ability groupings rather than dynamic and responsive instructional design (51).

In contrast, developed education systems demonstrate more advanced implementation models. In countries such as the United States, Australia, and across Europe, differentiated instruction is supported by data-driven assessment systems, digital technologies (e.g., wearable devices and AI-supported tools), and structured professional supervision (66–69). These systems enable continuous monitoring and adaptive instruction, ensuring that differentiation becomes an integral component of teaching practice rather than an optional strategy.

Overall, the findings suggest that the gap between policy and practice in implementation is primarily driven by structural limitations, insufficient professional support, and the absence of practical implementation frameworks. Addressing this gap requires systemic interventions, including increased instructional time allocation, sustained teacher professional development, and the provision of contextually adaptable teaching resources.

### Evaluating differentiated instruction in physical education

The findings further indicate that the evaluation of differentiated instruction remains predominantly within the *low to moderate* categories, with 78% of teachers falling into these levels and a mean score of 42.7 out of 60. This suggests that assessment practices responsive to individual student differences are not yet systematically implemented. Qualitative data reinforce this conclusion, revealing that most teachers continue to rely on uniform summative assessments, despite emerging efforts to incorporate more flexible approaches such as holistic rubrics and observational feedback.

From a conceptual standpoint, assessment within differentiated instruction should be grounded in principles of fairness, authenticity, and inclusivity (1). This entails the use of diverse formative strategies, including observational records, self-assessment, reflective journals, and portfolio-based evaluation (70). In physical education, such assessment must encompass cognitive, affective, and psychomotor domains in a balanced manner. Although the Merdeka Curriculum explicitly promotes diagnostic and formative assessment, the findings suggest that these principles have not yet been fully internalised in practice.

The persistence of conventional assessment practices can be attributed to several key factors. First, administrative and workload pressures limit teachers’ capacity to design and implement differentiated assessment instruments. The development of rubrics, documentation of individual progress, and provision of personalised feedback require substantial time and effort, which are often constrained within existing workload structures.

Second, limited assessment literacy among teachers restricts the effective application of formative and differentiated assessment strategies. Without adequate training and exemplars, teachers may lack the confidence and competence to move beyond standardised testing practices.

Third, infrastructural and technological limitations, particularly in eastern regions, hinder the documentation and monitoring of student progress. As a result, assessment practices tend to rely on informal and non-standardised methods, which, while contextually adaptive, reduce consistency and comparability.

These challenges are not unique to Indonesia. Studies from Australia and France indicate that differentiated assessment is frequently reduced to uniform summative testing that does not adequately capture learner diversity (67, 70). Similarly, research in the Philippines, Nepal, and the Maldives shows that, despite policy support for formative assessment, implementation remains limited and often constrained to simplified categorisations of student ability (51, 71–73). In Ethiopia, systemic barriers such as large class sizes, excessive workloads, and limited training further impede the adoption of differentiated assessment practices (52).

In contrast, developed education systems demonstrate more advanced and integrated approaches. In the United States, differentiated assessment is supported by digital tools, real-time feedback systems, and structured professional supervision, enabling continuous and personalised evaluation (74). In Ukraine, digital-based formative assessment systems incorporate diagnostic tools, self-reflection modules, and data visualisation platforms to support teacher development and student monitoring (75). These systems illustrate how technological and institutional support can transform assessment into a dynamic and responsive process.

The findings of this study therefore highlight a critical *implementation gap* between assessment policy and classroom practice. While the Merdeka Curriculum provides a strong conceptual foundation, its enactment is constrained by limited teacher readiness, insufficient resources, and the absence of practical implementation frameworks.

To address these challenges, several policy directions are recommended. First, the development of adaptive and context-sensitive assessment tools is essential, including simplified rubrics, observational templates, and accessible digital platforms. Second, continuous professional development should focus on enhancing teachers' assessment literacy, particularly in formative and differentiated evaluation strategies. Third, collaborative assessment practices involving teachers, school leaders, and parents should be strengthened to ensure that evaluation reflects meaningful learning processes rather than administrative compliance.

In sum, transforming evaluation practices within differentiated instruction requires not only technical improvements but also systemic changes that align policy, teacher capacity, and institutional support. Through such efforts, assessment can evolve into a core mechanism for promoting equity, inclusivity, and quality in physical education.

## Conclusion

This study reveals that the implementation of differentiated instruction in Indonesian primary school physical education remains largely within the *low to moderate* range, particularly in planning, implementation, and evaluation. While teachers demonstrate an emerging conceptual understanding, classroom practices remain inconsistent and only a minority apply contextually responsive strategies. The findings highlight a persistent gap between policy expectations under the Merdeka Curriculum and actual practice. This gap is driven by structural constraints, such as limited instructional time, inadequate resources, and uneven access to professional development, as well as regional disparities between western and eastern Indonesia. This study contributes to the literature by showing that the effectiveness of differentiated instruction is not solely determined by teacher competence, but is strongly influenced by contextual, socio-cultural, and systemic factors. Policy implications include the need to strengthen instructional support, enhance teacher training with context-sensitive approaches, and promote school-level collaboration to support practical implementation. However, this study is subject to several limitations that should be acknowledged. First, the use of self-reported data may introduce response bias, as teachers may overestimate their understanding and implementation of differentiated instruction due to social desirability and professional expectations. Second, the sampling strategy may have introduced selection bias. Participants were recruited through a national professional workshop, which is likely to attract teachers who are more professionally active, motivated, and exposed to recent educational reforms such as the Merdeka Curriculum. As a result, the findings may overestimate the overall level of teacher readiness and implementation of differentiated instruction compared to the broader population of primary school teachers in Indonesia. Third, the study was conducted within a limited timeframe, and the distribution of samples across provinces was not fully proportional, which may affect the generalisability of the findings. Therefore, the results should be interpreted as indicative of broader trends rather than as fully representative of national conditions.

## Data Availability

The raw data supporting the conclusions of this article will be made available by the authors, without undue reservation.

## References

[1] Tomlinson CA. Differentiated instruction in rural school contexts. In: Azano AP Callahan CM editors. Gifted Education in Rural Schools. New York: Routledge (2021). p. 79–90. 10.4324/9781003017004-11

[2] Uthus M Qvortrup A. Lessons learned from Norway: a values-based formulation of inclusive education. Eur J Spec Needs Educ. (2024) 40(2):244–58. 10.1080/08856257.2024.2354603

[3] Jager L Denessen E Cillessen A Meijer PC. Capturing instructional differentiation in educational research: investigating opportunities and challenges. Educ Res. (2022) 64(2):224–41. 10.1080/00131881.2022.2063751

[4] Ydo Y. Physical literacy on the global agenda. Prospects. (2021) 50(1–2):1–3. 10.1007/s11125-020-09524-8

[5] Bailey R Glibo I Koenen K Samsudin N. What is physical literacy? An international review and analysis of definitions. Kinesiol Rev. (2023) 12(3):247–60. 10.1123/kr.2023-0003

[6] Wintle J. Physical education and physical activity promotion: lifestyle sports as meaningful experiences. Educ Sci. (2022) 12(3):181. 10.3390/educsci12030181

[7] Haelermans C. The effects of group differentiation by students’ learning strategies. Instr Sci. (2022) 50(2):223–50. 10.1007/s11251-021-09575-0

[8] Ningsih AG. Exploring the impact of adaptive real-time quiz platforms with differentiated learning features on student engagement and learning outcomes: a mixed-methods approach. Int J Inf Educ Technol. (2025) 15(6):1261–76. 10.18178/ijiet.2025.15.6.2329

[9] Elumalai G Chinanapan K Choeibuakaew W Iqbal DR Abadi FH. Can model-based approach in physical education improve physical fitness, academic performance, and enjoyment among pupils? A systematic literature review. Int J Hum Mov Sports Sci. (2022) 10(4A):21–8. 10.13189/saj.2022.101304

[10] Dudley D Mackenzie E Van Bergen P Cairney J Barnett L. What drives quality physical education? A systematic review and meta-analysis of learning and development effects from physical education-based interventions. Front Psychol. (2022) 13:799330. 10.3389/fpsyg.2022.799330 35846697 PMC9280720

[11] Heidorn B Mosier B. Differentiation for student learning in physical education. Strategies. (2019) 32(4):40–4. 10.1080/08924562.2019.1608737

[12] Breed R Lindsay R Kittel A Spittle M. Content and quality of comparative tactical game-centered approaches in physical education: a systematic review. Rev Educ Res. (2024) 95:293–336. 10.3102/00346543241227236

[13] Teraoka E Ferreira HJ Kirk D Bardid F. Affective learning in physical education: a systematic review. J Teach Phys Educ. (2020) 40(3):460–73. 10.1123/jtpe.2019-0164

[14] Shen Y Shao W. Influence of hybrid pedagogical models on learning outcomes in physical education: a systematic literature review. Int J Environ Res Public Health. (2022) 19(15):9673. 10.3390/ijerph19159673 35955027 PMC9368380

[15] Akbaruddin A Suherman WS Komari A Hambali S Saputra W Permana MF. The utilization of differentiated learning in improving physical fitness and active lifestyle of junior high school students: literature review in physical education. Pamukkale J Sport Sci. (2024) 15:561–77. 10.54141/psbd.1512748

[16] Hasanah E Suyatno S Maryani I Badar MIA Fitria Y Patmasari L. Conceptual model of differentiated-instruction (DI) based on teachers’ experiences in Indonesia. Educ Sci. (2022) 12(10):650. 10.3390/educsci12100650

[17] Gustian U Saputra DR Rakhmat C Yustiana YR Primayanti I. Physical education and its scope: a literature review of empirical studies with a holistic perspective teaching practices in Indonesia. Indones J Phys Educ Sport Sci. (2024) 4(2):171–86. 10.52188/ijpess.v4i2.729

[18] Maulana FI Heynoek FP. Pendekatan berdiferensiasi pada Pelajaran Pendidikan Jasmani Olahraga dan Kesehatan. J Innov Teach Prof. (2024) 2(3):320–8. 10.17977/um084v2i32024p320-328

[19] Utaminingsih ES Wuriningsih FR Intania BY Idammatussilmi I. Empowering sustainable education through differentiated learning: a systematic review in primary school. J Ilm Profesi Pendidik. (2025) 10(1):63–71. 10.29303/jipp.v10i1.3077

[20] Taek V. Teachers’ perspectives on differentiated learning in the independent curriculum: context Indonesia. Panicgogy Int J. (2024) 2(1):39–47. 10.59965/pij.v2i1.149

[21] Sari DM Maulida F Khoirunnisa JPN Ummah SK Admoko S. A literature review of the implementation of differentiated learning in Indonesian education units. J Ilm Pendidik Fis. (2023) 7(2):250. 10.20527/jipf.v7i2.8429

[22] Ramadhini NAJ Sukmawan S. Refleksi Diri Guru Praktikan dalam Proses Pembelajaran Berdiferensiasi Mata Pelajaran Bahasa Indonesia. ASATIZA J Pendidik. (2024) 5(2):131–43. 10.46963/asatiza.v5i2.1785

[23] Verianti NG Muhyi NM Ismaryati N Hanafi NM Hakim NL Suharti N. Survei Pembelajaran Berdifferensiasi Pada Mata Pelajaran PJOK di Tingkat Sekolah Dasar: Tingkat Pemahaman Sampai Penerapan. J STAND Sports Teach Dev. (2024) 5(1):65–74. 10.36456/j-stand.v5i1.9594

[24] Citra Y Nopembri S Susanto SPY. Evaluation study of physical education learning in sports and health in special schools in Yogyakarta. Int J Multidiscipl Res Anal. (2024) 7(1):411–6. 10.47191/ijmra/v7-i01-49

[25] Taryatman T Rahim A. Strategi Pembelajaran Pendidikan Jasmani Di Sekolah Dasar Inklusif Kota Yogyakarta. Taman Cendekia. (2018) 2(2):212–22. 10.30738/tc.v2i2.3143

[26] Kaloka P Purwanto S Wibowo Y. Teachers administration implementation of physical education of adapted school in yogyakarta. Proceedings of the 3rd Yogyakarta International Seminar on Health, Physical Education, and Sport Science in Conjunction With the 2nd Conference on Interdisciplinary Approach in Sports (2019). p. 330–5. 10.5220/0009785903300335

[27] Wipulanusat W Panuwatwanich K Stewart RA Sunkpho J. Applying mixed methods sequential explanatory design to innovation management. In: Panuwatwanich K Ko CH, editors. Lecture Notes in Mechanical Engineering. Singapore: Springer (2020). p. 485–95. 10.1007/978-981-15-1910-9_40

[28] Jung SY Moon H Park DSM Sung S Jung H. Nurses’ burden of elimination care: sequential explanatory mixed-methods design. Int J Gen Med. (2023) 16:4067–76. 10.2147/ijgm.s424424 37700744 PMC10493134

[29] Wang Y Harris JJ. Understanding engineering students’ connection making: a sequential explanatory mixed methods study. 2021 IEEE Frontiers in Education Conference (FIE) (2023). p. 1–7

[30] Patton MQ. Qualitative research & evaluation methods: integrating theory and practice. In Medical Entomology and Zoology. Available online at: http://ci.nii.ac.jp/ncid/BB18275167 (Accessed May 23, 2024).

[31] Creswell JW. Designing and Conducting Mixed Methods Research, 3rd ed. Thousand Oaks, CA: SAGE (2018).

[32] Coubergs C Struyven K Vanthournout G Engels N. Measuring teachers’ perceptions about differentiated instruction: the DI-quest instrument and model. Stud Educ Eval. (2017) 53:41–54. 10.1016/j.stueduc.2017.02.004

[33] Koo M Yang S. Likert-type scale. Encyclopedia. (2025) 5(1):18. 10.3390/encyclopedia5010018

[34] Emerson RW. Likert scales. J Vis Impair Blind. (2017) 111(5):488. 10.1177/0145482x1711100511

[35] Roberts JS Wedell DH Laughlin JE. Heightened sensitivity of likert attitude scales to restriction of sample range. American Educational Research Association, Annual Meeting of the American Educational Research Association (1998).

[36] Dorji T Nima P. Evaluating content validity of an instrument to assess teachers’ practice of differentiated instruction (IATPDI). Bhutan J Res Dev. (2021) 10(2):75–96. 10.17102/bjrd.rub.10.2.006

[37] Zhao Y Bian L Yang J. Data.xlsx [Dataset]. *Figshare* (2022). 10.6084/m9.figshare.19721743

[38] Alrashed A. The influence of using statistical software on educational decision making in Jordan private universities. 2022 International Conference on Decision Aid Sciences and Applications (DASA) (2024). p. 1–5

[39] Braun V Clarke V. Using thematic analysis in psychology. Qual Res Psychol. (2006) 3(2):77–101. 10.1191/1478088706qp063oa

[40] Faulkner SL Atkinson JD editors. Qualitative Methods in Communication and Media. Oxford: Oxford University Press (2023). 10.1093/oso/9780190944056.001.0001

[41] Pratt MG. General coding and analysis in qualitative research. Oxford Research Encyclopedia of Psychology (2023).

[42] Lovell K Callaghan P Brooks H Bee P editors. A Research Handbook for Patient and Public Involvement Researchers. Manchester: Manchester University Press (2018). 10.7765/9781526136527

[43] Halim A Iskandar N Ansari A Halim NM. A study on how the Merdeka curriculum promotes multilingualism in Indonesian ELT classrooms. XLinguae. (2024) 17(2):107–21. 10.18355/xl.2024.17.02.07

[44] Rahmah L Purwanta E Wijayanti W Suhardiman S. Navigating the curriculum landscape: the impact of curriculum 2013 and Merdeka curriculum on teachers’ and students’ learning outcomes in Indonesia. J Ecohumanism. (2024) 3(6):917–31. 10.62754/joe.v3i6.4061

[45] Samsudi S Supraptono E Utanto Y Rohman S Djafar T. Unraveling the Merdeka curriculum: exploring differentiated instruction’s impact on student learning. J Ilm Peuradeun. (2024) 12(2):517–38. 10.26811/peuradeun.v12i2.1131

[46] Yavuz AC. The effects of differentiated instruction on Turkish students’ L2 achievement, and student and teacher perceptions. Eur J Appl Linguist. (2020) 6(2):313–35. 10.32601/ejal.776002

[47] Uçarkuş E. Teachers’ perspectives on differentiated instruction in Turkey: a meta-synthesis study. Trakya Eğitim Dergisi. (2024) 14(1):442–58. 10.24315/tred.1392263

[48] Nychkalo N Lukianova L Bidyuk N Tretko V Skyba K. Didactic aspects of teachers’ training for differentiated instruction in modern school practice in Ukraine. Int J Learn Teach Educ Res. (2020) 19(9):145–59. 10.26803/ijlter.19.9.8

[49] Bi M Struyven K Zhu C. Differentiated instruction in Chinese primary and secondary schools: a systematic literature review. Res Sq. (2021). 10.21203/rs.3.rs-207311/v1

[50] Goyibova N Muslimov N Sabirova G Kadirova N Samatova B. Differentiation approach in education: tailoring instruction for diverse learner needs. MethodsX. (2025) 14:103163. 10.1016/j.mex.2025.103163 39897653 PMC11786651

[51] Adam M Porta T. Primary teachers’ perceptions of differentiated instruction: insights from Maldivian classrooms. Educ. (2025) 3-13:1–13. 10.1080/03004279.2025.2473412

[52] Mengistie SM. Primary school teachers’ knowledge, attitude and practice of differentiated instruction. Int J Curric Instr. (2020) 12(1):98–114. Available online at: http://ijci.wcci-international.org/index.php/IJCI/article/download/258/154

[53] Fauziah FN Saddhono K Suryanto E. Implementation of local wisdom-based Indonesian learning to strengthen Pancasila student profiles (P5): case studies in vocational high schools. J Curric Teach. (2023) 12(6):283. 10.5430/jct.v12n6p283

[54] Tapung M. Evaluating the key success factors of “Merdeka” curriculum: evidence from east Nusa Tenggara, Indonesia. J Curric Teach. (2025) 14(2):88. 10.5430/jct.v14n2p88

[55] Mihajlovic C. Teachers’ perceptions of the Finnish national curriculum and inclusive practices of physical education. Curric Stud Health Phys Educ. (2019) 10(3):247–61. 10.1080/25742981.2019.1627670

[56] Suwalska A. Transversal competencies in physical education in the Finnish national core curriculum 2014 for basic education. Ann Univ Mariae Curie-Skłodowska J Paedagog-Psychol. (2021) 34(2):83–94. 10.17951/j.2021.34.2.83-94

[57] Afdal HW. Policy making processes with respect to teacher education in Finland and Norway. High Educ. (2012) 65(2):167–80. 10.1007/s10734-012-9527-2

[58] Volmari S. Constellation of trajectories and fast policy worlds: a spatiotemporal reading of experts’ positions and social encounters in Finland’s and Norway’s recent curriculum reforms. Nord J Stud Educ Policy. (2022) 8(3):184–95. 10.1080/20020317.2022.2151105

[59] Burnett C. Addressing challenges of PE in South African public schools. S Afr J Res Sport Phys Educ Recreat. (2020) 42(2):15–30. Available online at: https://www.ajol.info/index.php/sajrs/article/view/200105

[60] Colquitt G Pritchard T Johnson C McCollum S. Differentiating instruction in physical education: personalization of learning. J Phys Educ Recreat Dance. (2017) 88(7):44–50. 10.1080/07303084.2017.1340205

[61] Zerai D Eskelä-Haapanen S Posti-Ahokas H Vehkakoski T. The meanings of differentiated instruction in the narratives of Eritrean teachers. Pedagogy Cult Soc. (2021) 31(3):419–37. 10.1080/14681366.2021.1914712

[62] Hameed M Dilshad M Rasool T. Use of differentiated instruction at special education schools: teachers’ perspective. Pak J Humanit Soc Sci. (2024) 12(2):2206–17. 10.52131/pjhss.2024.v12i2.2401

[63] Fomunyam KG Khoza SB editors. Curriculum Theory, Curriculum Theorising, and the Theoriser: The African Theorising Perspective. Leiden: Brill (2021). Available online at: https://brill.com/view/title/59575

[64] Van Munster MA Lieberman LJ Grenier MA. Universal design for learning and differentiated instruction in physical education. Adapt Phys Activ Q. (2019) 36(3):359–77. 10.1123/apaq.2018-0145 31155914

[65] Braga L Tracy JF Taliaferro AR. Physical activity programs in higher education: modifying net/wall games to include individuals with disabilities. J Phys Educ Recreat Dance. (2014) 86(1):16–22. 10.1080/07303084.2014.978417

[66] Borthwick AC Anderson CL Finsness ES Foulger TS. Special article personal wearable technologies in education: value or villain? J Digit Learn Teach Educ. (2015) 31(3):85–92. 10.1080/21532974.2015.1021982

[67] Whipp P Taggart A Jackson B. Differentiation in outcome-focused physical education: pedagogical rhetoric and reality. Phys Educ Sport Pedagogy. (2012) 19(4):370–82. 10.1080/17408989.2012.754001

[68] Runge I Lazarides R Rubach C Richter D Scheiter K. Teacher-reported instructional quality in the context of technology-enhanced teaching: the role of teachers’ digital competence-related beliefs in empowering learners. Comput Educ. (2023) 198:104761. 10.1016/j.compedu.2023.104761

[69] Xue J Zhang J. A data-driven evaluation of student health impacts in teaching higher education physical education and health programs. Appl Math Nonlinear Sci. (2024) 9(1):1–14. 10.2478/amns-2024-3490

[70] Mastagli M Malini D Hainaut J Van Hoye A Bolmont B. Summative assessment versus formative assessment: an ecological study of physical education by analyzing state-anxiety and shot-put performance among French high school students. J Phys Educ Sport. (2020) 2020(3):2220–9. 10.7752/jpes.2020.s3298

[71] Griffin P Cagasan L Care E Vista A Nava F. Formative assessment policy and its enactment in the Philippines. In: Laveault D Allal L, editors. The Enabling Power of Assessment. Cham: Springer (2016). p. 75–92. 10.1007/978-3-319-39211-0_5

[72] Van Der Kleij FM Cumming JJ Looney A. Policy expectations and support for teacher formative assessment in Australian education reform. Assess Educ Princ Policy Pract. (2017) 25(6):620–37. 10.1080/0969594x.2017.1374924

[73] Prajapati PL. Policy and practice gap in continuous assessment system provisioned under integrated curriculum. Ganeshman Darpan. (2024) 9(1):33–40. 10.3126/gd.v9i1.68543

[74] Baumoel M Schmidlein R. Differentiation: a teacher’s perspective. Strategies. (2023) 36(2):18–24. 10.1080/08924562.2023.2174227

[75] Morze N Buinytska O Varchenko-Trotsenko L Terletska T. Differentiated system for digital professional development of university teachers. Proceedings of the 2nd Myroslav I. Zhaldak Symposium on Advances in Educational Technology - AET (2021). p. 35–45

