# Correction: Differentiated instruction in Indonesian primary school physical education: a mixed-methods study

**DOI:** 10.3389/fspor.2026.1901023

**Published:** 2026-06-29

**Authors:** Frontiers Production Office

**Affiliations:** Frontiers Media SA, Lausanne, Switzerland

**Keywords:** differentiated instruction, elementary school students, instructional planning, physical education teacher, teaching implementation

An incorrect **Funding** statement was provided. The correct statement reads:

“The author(s) declared that financial support was received for this work and/or its publication. Financial support was received for the publication of this article through the Enhancing Quality Education for International University Impacts and Recognition (EQUITY) Program – THE Impact Ranking 2025–2026 provided by the International Office of Yogyakarta State University (UNY), Indonesia. The APC support was granted under official letter No. 314/UN34.36/KI/2025 issued by the International Office, Yogyakarta State University.”

The **Acknowledgments** statement was missing. The statement reads:

“The authors would like to express their sincere gratitude to the Rector of Yogyakarta State University (UNY), the Dean of the Faculty of Sport and Health Sciences (FIKK) UNY, and the Indonesian Sports Professor Association (AKPORI) for their continuous academic support and research facilitation throughout this study. The authors also acknowledge the financial support for the Article Processing Charge (APC) provided through the Enhancing Quality Education for International University Impacts and Recognition (EQUITY) Program – THE Impact Ranking 2025–2026 administered by the International Office of Yogyakarta State University under Letter No. 314/UN34.36/KI/2025.”

The original version of this article has been updated.

